# LINX^®^, a novel treatment for patients with refractory asthma complicated by gastroesophageal reflux disease: a case report

**DOI:** 10.1186/s13256-016-0887-6

**Published:** 2016-05-24

**Authors:** Narin Sriratanaviriyakul, Celeste Kivler, Tamas J. Vidovszky, Ken Y. Yoneda, Nicholas J. Kenyon, Susan Murin, Samuel Louie

**Affiliations:** 1 Division of Pulmonary, Critical Care, and Sleep Medicine, Department of Internal Medicine, University of California, Davis, Sacramento, CA USA; 2 VA Northern California Health Care System, Mather, CA USA; 3 Department of Internal Medicine, The Queen’s Medical Center, 1301 Punchbowl St, Honolulu, HI 96813 USA; 4 Department of Respiratory Care, University of California, Davis, 4150 V Street, Suite 3100, Sacramento, CA 95817 USA; 5 Department of Surgery, University of California, Davis, 4150 V Street, Suite 3100, Sacramento, CA 95817 USA

**Keywords:** Asthma, Fundoplication, GERD, LINX, Reflux

## Abstract

**Background:**

Gastroesophageal reflux disease is one of the most common comorbidities in patients with asthma. Gastroesophageal reflux disease can be linked to difficult-to-control asthma. Current management includes gastric acid suppression therapy and surgical antireflux procedures. The LINX® procedure is a novel surgical treatment for patients with gastroesophageal reflux disease refractory to medical therapy. To the best of our knowledge, we report the first case of successful treatment of refractory asthma secondary to gastroesophageal reflux disease using the LINX® procedure.

**Case presentation:**

Our patient was a 22-year-old white woman who met the American Thoracic Society criteria for refractory asthma that had remained poorly controlled for 5 years despite progressive escalation to step 6 treatment as recommended by National Institutes of Health-National Asthma Education and Prevention Program guidelines, including high-dose oral corticosteroids, high-dose inhaled corticosteroid plus long-acting β_2_-agonist, leukotriene receptor antagonist, and monthly omalizumab. Separate trials with azithromycin therapy and roflumilast did not improve her asthma control, nor did bronchial thermoplasty help. Additional consultations with two other university health systems left the patient with few treatment options for asthma, which included cyclophosphamide. Instead, the patient underwent a LINX® procedure after failure of maximal medical therapy for gastroesophageal reflux disease with the additional aim of improving asthma control. After she underwent LINX® treatment, her asthma improved dramatically and was no longer refractory. She had normal exhaled nitric oxide levels and loss of peripheral eosinophilia after LINX® treatment. Prednisone was discontinued without loss of asthma control. The only immediate adverse effects due to the LINX® procedure were bloating, nausea, and vomiting.

**Conclusions:**

LINX® is a viable alternative to the Nissen fundoplication procedure for the treatment of patients with gastroesophageal reflux disease and poorly controlled concomitant refractory asthma.

## Background

The prevalence of gastroesophageal reflux disease (GERD) is increased among patients with asthma. Up to 80 % of patients with asthma report GERD symptoms [1, 2]. Refractory asthma accounts for 5–10 % cases of asthma, and control of symptoms may be unattainable because of GERD and other comorbidities. A pathophysiological link between the two has been suggested and studied; however, their exact correlation to one another has not been fully established.

The National Asthma Education and Prevention Program (NAEPP) Expert Panel recommends that GERD should be treated only in patients with asthma and symptomatic GERD. Symptoms can be absent in more than 60 % of patients confirmed to have GERD by esophageal manometry and 24-h esophageal pH testing [3]. The two primary management options are long-term medical acid suppression therapy with proton pump inhibitors (PPIs) and/or H_2_ receptor antagonists, and surgical Nissen fundoplication [4]. The latter is recommended when maximal medical management for GERD fails. Sphincter augmentation with the LINX® Reflux Management System (Torax Medical, Shoreview, MN, USA) is a novel alternative procedure for GERD treatment. We report our experience with a patient whose asthma was very poorly controlled despite high-intensity asthma treatment that included systemic corticosteroid, omalizumab, and bronchial thermoplasty, and who benefited from LINX® treatment.

## Case presentation

Our patient was a 22-year-old white woman who met the criteria of refractory asthma as determined by an American Thoracic Society workshop in 2000. Her asthma remained poorly controlled despite progressive escalation therapy as recommended in NAEPP guidelines, including daily high-dose inhaled corticosteroids plus a long-acting β_2_-agonist, a leukotriene receptor antagonist, inhaled anticholinergics, and daily high-dose oral corticosteroids. Her asthma symptoms were not controlled with additional treatments with omalizumab, macrolide therapy, and U.S. Food and Drug Administration off-label used of roflumilast. The patient completed three sessions of bronchial thermoplasty treatment, also without benefit. She later consulted two other university health systems, whose clinicians agreed with her diagnosis of refractory asthma and recommended cyclophosphamide and anti-interleukin-13 as alternative treatments.

The patient’s physical examination was remarkable only for wheezing during acute exacerbations. Pertinent laboratory results, radiographic findings, and investigations at three university health systems included a pulmonary function test with moderate obstruction and significant bronchodilator response. Her methacholine challenge was positive for bronchial hyperactivity. Her complete blood count was unremarkable aside from peripheral blood eosinophilia of 7.2 % or 700 cells/mm^3^. Her exhaled nitric oxide was consistently elevated above 100 parts per billion (ppb), reaching a peak of 266 ppb just before she underwent the LINX® procedure. Her antinuclear antibody titer was 1:40. Her test results for antineutrophil cytoplasmic antibody, anticentromere, anti-Ro/SSA and anti-La/SSB antigen, rheumatoid factor, and anti-cyclic citrullinated peptide antibodies were all negative. Her α_1_-antitrypsin level was 150 mg/mL. Her immunoglobulin (Ig) levels (kU/L) before omalizumab treatment were IgE 929, IgG 857, IgM 118, and IgA 110. Her *Aspergillus* precipitin results were negative, and her radioallergosorbent test panel result was positive for perennial aeroallergens. Her skin prick test results for the Northern California aeroallergen panel were positive for multiple trees, grasses, weeds, and cat.

High-resolution computed tomography of her chest was remarkable for bronchial wall thickening and air trapping without evidence of bronchiectasis or tracheomalacia. Her bronchoscopy result was significant for severe, diffuse erythema throughout the lower respiratory tract. Her bronchoalveolar lavage specimens were unremarkable in terms of cell counts, cytology, and cultures. The results of her endobronchial biopsy of the right lower lobe were consistent with asthma with severe airway epithelial injury and cell loss. Her steroid pharmacokinetics and pharmacodynamics were within normal limits.

The patient returned to our institution and complained of heartburn. She had been diagnosed with GERD and had been receiving therapy with a PPI, H_2_ antagonist, and metoclopramide for 2 years. Her 24-h probe monitoring showed severe acid exposure while in supine position. We concluded that she did not have adequate control of her GERD despite medical management. A surgical antireflux procedure with options of fundoplication or the LINX® procedure was considered. The patient underwent the LINX® procedure. Following the procedure, her asthma control improved dramatically. Her exhaled nitric oxide fell from 266 ppb to 17 ppb 1 month after surgery, and her peripheral eosinophilia returned to normal levels. Prednisone was discontinued 4 months after surgery. She lost weight, and her body mass index decreased from 36.4 kg/m^2^ preoperatively to 34.6 kg/m^2^ at her 2-week postoperative follow-up. The only immediate adverse effects she experienced due to the LINX® procedure were bloating, nausea, and vomiting, all of which have since subsided.

## Discussion

GERD is one of the most common comorbidities in patients with asthma. GERD can contribute to and worsen asthma symptoms and asthma control. Current management of symptomatic GERD includes acid suppression therapy and surgical antireflux procedures. The connection between GERD and asthma remains controversial, but an association seems to exist [1–3]. Asthma appears to be exacerbated by GERD, and a significant portion of patients report improvement of asthma symptoms following better control of GERD. Conversely, asthma treatment such as albuterol and even corticosteroids may worsen GERD, purportedly by reducing lower esophageal sphincter (LES) tone [5, 6].

GERD management can be categorized into medical management or surgical management or both. Acid suppression treatment with PPIs and/or H_2_ antagonists is an effective first-line therapy in most cases, and this strategy seems to improve respiratory symptoms and reduce the need for asthma medication, with varying degrees of success [7].

Pharmacological therapy fails in a significant proportion of patients with asthma and GERD, even after years of clinical trials. Despite treatment, GERD may progress and lead to complications such as strictures and Barrett’s esophagus [8]. It is believed that acid-inhibiting medication may reduce or neutralize the acid but that reflux can continue because of an incompetent LES. The conventional surgical approach to the latter problem is surgical fundoplication to restore a physical barrier to reflux. Recent literature suggests that laparoscopic fundoplication surgery may be more effective than medical management for GERD, at least in the short to medium term [9]. However, fundoplication is associated with complications and a slight mortality risk; in addition, it alters gastric anatomy. For these reasons, in the United States, less than 1 % of patients with GERD undergo Nissen fundoplication [10].

**Fig. 1:**
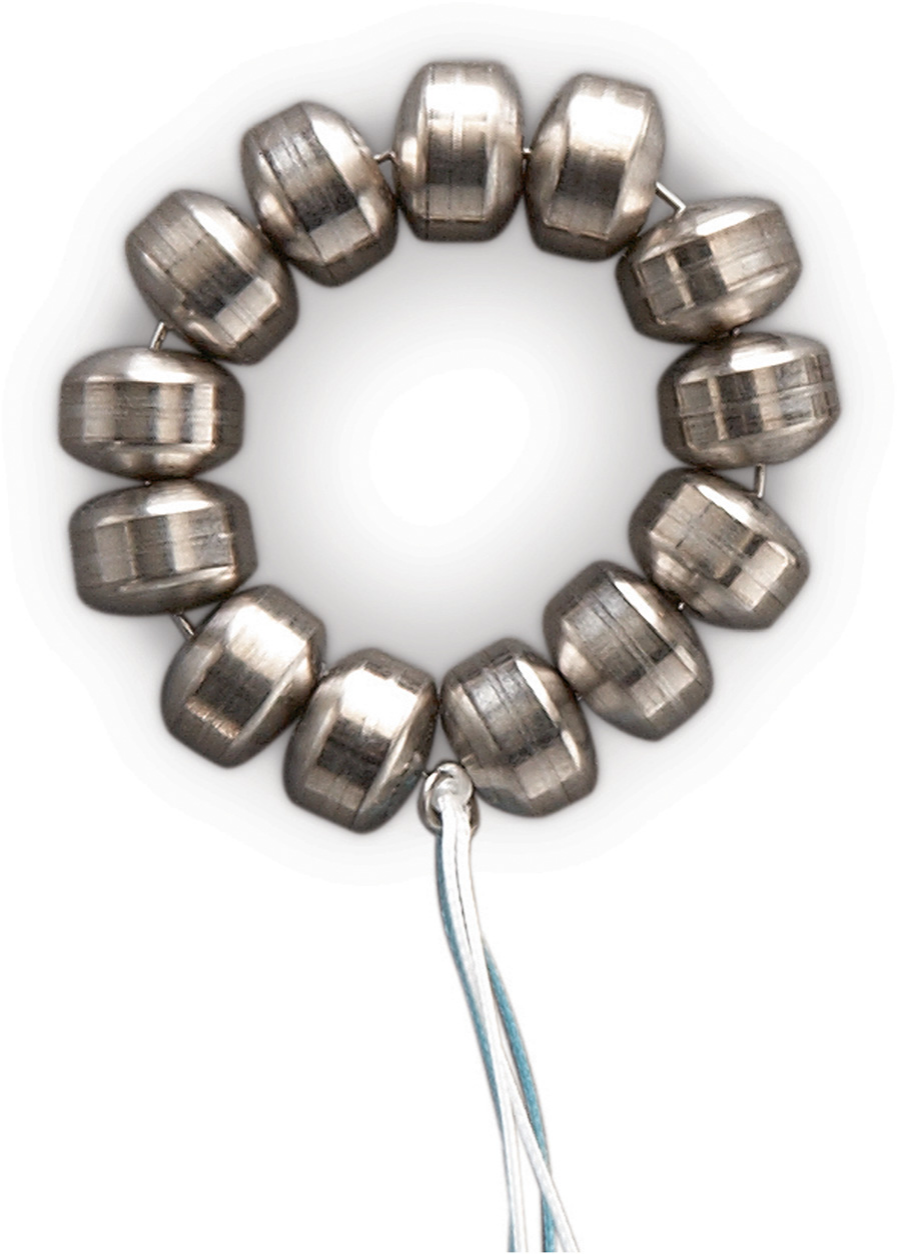
The LINX® Reflux Management System. The device is composed of a series of titanium beads, each with a magnetic core, connected together with independent titanium wires to form a ring shape

**Fig. 2:**
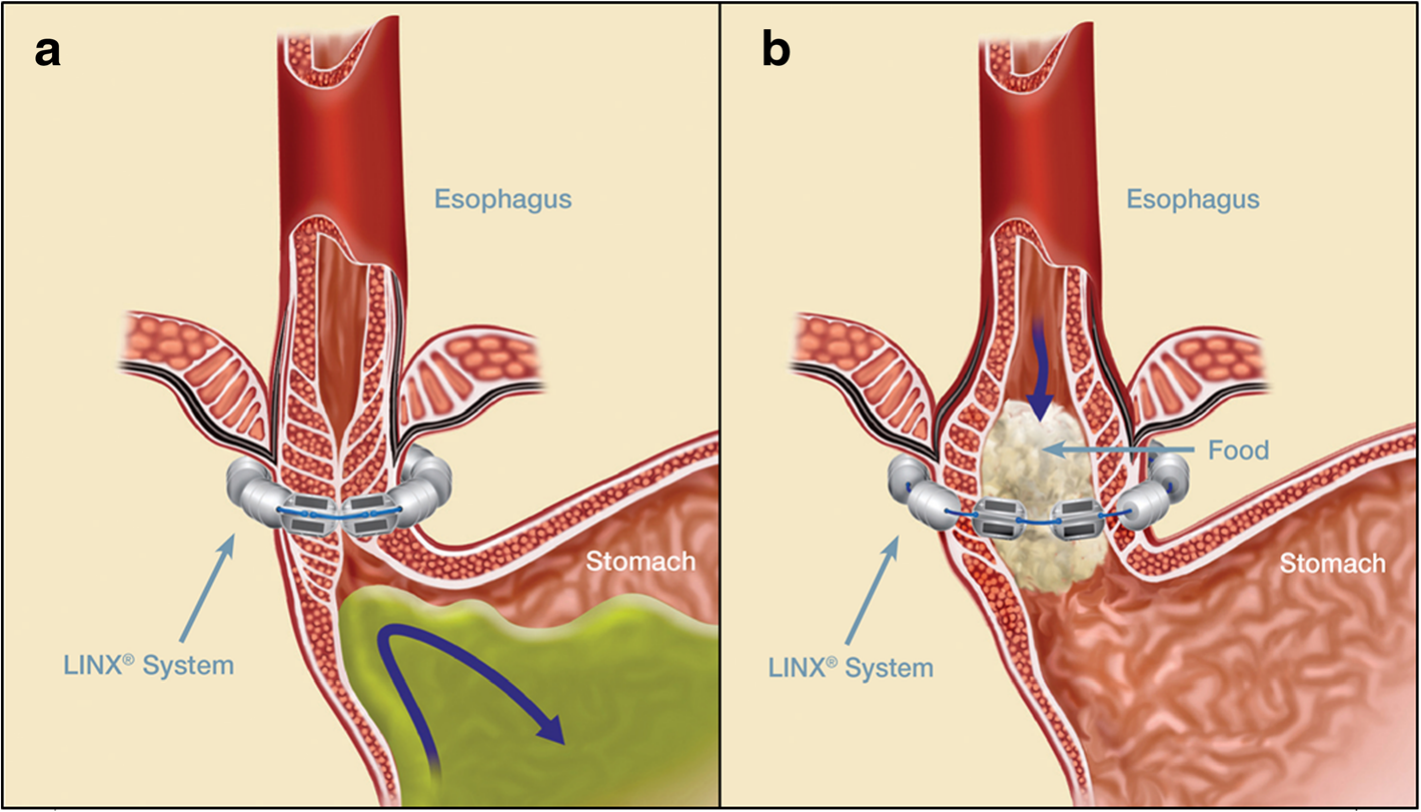
**a** The LINX® Reflux Management System is designed to help the lower esophageal sphincter resist opening to gastric pressures. **b** The LINX® Reflux Management System is designed to expand to allow for normal swallowing

The LINX® Reflux Management System was developed to address the “therapy gap” between conventional medical acid suppression therapy and fundoplication surgery [4]. The aim of this procedure is to restore a physiological equivalent of an LES. The device is composed of a series of titanium beads, each with a magnetic core, connected together with independent titanium wires to form a ring shape (Fig. 1). It serves to prevent the backward flow of stomach contents (Fig. 2). It is implanted laparoscopically at the LES. Upon swallowing by the patient, the device expands to let the contents pass. Once the food passes though the LES, the device returns to its resting state. Current evidence supports its treatment effect and safety profile [11]. The most common adverse event reported is dysphagia (43 % of patients), which is mostly mild and self-limited. In the event of a serious adverse event, the LINX® device is retrievable. The LINX® device is considered magnetic resonance imaging (MRI)-conditional, as it is safe with MRI systems up to 1.5 T.

The case of our patient illustrates the importance of recognizing and managing comorbidities in patients with difficult-to-control asthma. Our patient was referred for surgery after her GERD was refractory to maximal medical management as evidenced by 24-h pH probe testing. The LINX® was chosen over fundoplication because there is evidence that the LINX® minimally alters gastric anatomy while augmenting the LES, thereby preserving the option of fundoplication in the future should LINX® treatment fail. The improvement in our patient’s asthma symptoms and biomarkers achieved immediately following the procedure suggest that GERD contributed significantly to her poor asthma control. To our knowledge, this is the first case of successful treatment of refractory asthma secondary to GERD with the LINX® Reflux Management System.

## Conclusions

LINX® is a viable alternative to Nissen fundoplication as a treatment option for patients with poorly controlled GERD who have concomitant refractory asthma.

## Abbreviations

GERD: gastroesophageal reflux disease
Ig: immunoglobulin
LES: lower esophageal sphincter
MRI: magnetic resonance imaging
NAEPP: National Asthma Education and Prevention Program
ppb: parts per billion
PPI: proton pump inhibitor
